# Delineation of the Direct Contribution of Candida auris *ERG11* Mutations to Clinical Triazole Resistance

**DOI:** 10.1128/Spectrum.01585-21

**Published:** 2021-12-08

**Authors:** Jeffrey M. Rybak, Cheshta Sharma, Laura A. Doorley, Katherine S. Barker, Glen E. Palmer, P. David Rogers

**Affiliations:** a Department of Pharmacy and Pharmaceutical Sciences, St. Jude Children’s Research Hospital, Memphis, Tennessee, USA; b Department of Clinical Pharmacy and Translational Science, University of Tennessee College of Pharmacy, Memphis, Tennessee, USA; University of Michigan

**Keywords:** *Candida*, triazole, resistance, *ERG11*, CRISPR

## Abstract

Resistance to fluconazole is one of clinical characteristics most frequently challenging the treatment of invasive Candida auris infections, and is observed among >90% of all characterized clinical isolates. In this work, the native C. auris *ERG11* allele in a previously characterized fluconazole-susceptible clinical isolate was replaced with the *ERG11* alleles from three highly fluconazole-resistant clinical isolates (MIC ≥256 mg/L), encoding the amino acid substitutions VF125AL, Y132F, and K143R, using Cas9-ribonucleoprotein (RNP) mediated transformation system. Reciprocally, the *ERG11*^WT^ allele from the same fluconazole-susceptible clinical isolate, lacking any resistance-associated mutation, was introduced into a previously characterized fluconazole-resistant clinical isolate, replacing the native *ERG11*^K143R^ allele, using the same methods. The resulting collection of strains was subjected to comprehensive triazole susceptibility testing, and the direct impact each of these clinically-derived *ERG11* mutations on triazole MIC was determined. Introduction of each of the three mutant *ERG11* alleles was observed to increase fluconazole and voriconazole MIC by 8- to 16-fold. The MIC for the other clinically available triazoles were not significantly impacted by any *ERG11* mutation. In the fluconazole-resistant clinical isolate background, correction of the K143R encoding mutation led to a similar 16-fold decrease in fluconazole MIC, and 8-fold decrease in voriconazole MIC, while the MIC of other triazoles were minimally changed. Taken together, these findings demonstrate that mutations in C. auris *ERG11* significantly contribute to fluconazole and voriconazole resistance, but alone cannot explain the substantially elevated MIC observed among clinical isolates of C. auris.

**IMPORTANCE** Candida auris is an emerging multidrug-resistant and health care-associated pathogen of urgent clinical concern. The triazoles are the most widely prescribed antifungal agents worldwide and are commonly utilized for the treatment of invasive *Candida* infections. Greater than 90% of all C. auris clinical isolates are observed to be resistant to fluconazole, and nearly all fluconazole-resistant isolates of C. auris are found to have one of three mutations (encoding VF125AL, Y132F, or K143R) in the gene encoding the target of the triazoles, *ERG11*. However, the direct contribution of these mutations in *ERG11* to fluconazole resistance and the impact these mutations may have the susceptibility of the other triazoles remains unknown. The present study seeks to address this knowledge gap and potentially inform the future application the triazole antifungals for the treatment of infections caused by C. auris.

## INTRODUCTION

Among pathogenic fungi, the most commonly reported mechanism of resistance to the triazole class of antifungals is the acquisition of mutations in the gene encoding sterol-demethylase, the enzyme which these agents directly interact with and inhibit (1–3). Frequently reported in association with triazole resistance in both yeast and molds, mutations in sterol-demethylase have been observed to have variable and agent specific effects on the MICs of triazole antifungals, potentially as a result of agent-specific stabilizing interactions with sterol-demethylase residues (4, 5). In both Candida albicans and Aspergillus fumigatus, the mutation encoding a tyrosine to phenylalanine amino acid substitution at a specific residue localized to the sterol-demethylase catalytic domain (Y132F in C. albicans *CaERG11* and Y121F in A. fumigatus *Afcyp51A*) confers increased resistance to all triazoles except for posaconazole (4–7). Conversely, in both of these clinically important fungal pathogens, other sterol-demethylase mutations confer only agent specific increases in resistance. Thus, understanding the specific impact of such mutations on susceptibility to individual triazoles has significant clinical implications as such knowledge can aid in selection of optimal antifungal therapy and in the development of new sterol-demethylase inhibitors.

Candida auris is an emerging multi-drug resistant fungal pathogen of global concern (8–10). Prominent among the multitude of unique features contributing to the clinical significance of C. auris is the prevalence and high-degree of resistance to the most commonly prescribed antifungal agent, fluconazole. Testing hundreds of clinical C. auris clinical isolates, the Center for Disease Control and Prevention (CDC) has found the modal MIC of all C. auris isolates from the United States to be ≥256 mg/L with 90% of isolates possessing fluconazole MIC ≥32 mg/L (the tentative clinical breakpoint set forth by the CDC) (11).

Almost all fluconazole-resistant clinical isolates have been reported to possess mutations in *ERG11* (B9J08_001448), the C. auris gene encoding sterol-demethylase. Intriguingly, only three predominant mutations in *ERG11* have been identified, encoding the amino acid substitutions VF125AL (more commonly reported simply as F126L), Y132F, and K143R, and each of these mutations is strongly associated with specific genetic clades of C. auris (9). Namely, the VF125AL-encoding mutation is uniquely found among clinical isolates from Clade III (originally associated with South Africa), the Y132F-encoding mutation is found among clinical isolates from both Clade I and Clade IV (originally associated with South Asia and South America, respectively), and the K143R-encoding mutation is most notably found among clinical isolates from Clade I. However, individual isolates harboring the mutation encoding the K143R amino acid substitution have also been found to belong to Clade II (originally associated with East Asia) and Clade IV (12, 13). While clinical isolates with fluconazole MIC as low as 2 mg/L and harboring these mutations in *ERG11* have been reported, the predominance of isolates with these mutations have MIC well above the tentative breakpoints as set forth by the CDC.

Heterologous expression of C. auris *ERG11* alleles carrying the mutations leading to the Y132F and K143R substitutions on a low copy number episomal plasmid in Saccharomyces cerevisiae revealed that both mutations increase fluconazole resistance, but the specific contribution of these mutations to triazole resistance in C. auris has not been defined (14). It cannot currently be determined if the most frequently identified resistance-associated mutations in C. auris are solely responsible for the as high as 256-fold difference in fluconazole MIC among clinical isolates, and the impact that each of these mutations has on the MIC of the other clinically available triazoles is unknown. In this work, a Cas9-mediated transformation system is utilized to delineate the impact of each of the three most commonly encountered *ERG11* mutations (encoding the amino acid substitutions VF125AL, Y132F, and K143R) on triazole antifungal susceptibility directly in C. auris clinical isolates.

## RESULTS

In an effort to delineate the direct impact of each of the three clinically relevant mutations in the C. auris *ERG11* gene, a Cas9-mediated transformation system incorporating entire C. auris *ERG11* alleles of interest, the *SAT-FLP* cassette, 50 bases of downstream microhomology, and dual targeting Cas9-RNP was developed. Briefly, the wild type *ERG11* allele (*ERG11*^WT^) from the clinical isolate previously used to construct the Clade Ia genome assembly, AR0387 (also known as B8441), as well as the *ERG11* alleles from three highly fluconazole-resistant clinical isolates carrying the VF125AL, Y132F, and K143R-encoding mutations, were individually cloned into the pBSS2 plasmid which contains the *SAT-FLP* cassette. Notably, the *ERG11*^VF125AL^ and *ERG11*^Y132F^ alleles additionally include polymorphisms not associated with fluconazole resistance and common to clinical isolates of Clade III and IV, respectively, including fluconazole-susceptible isolates. Each of the four resulting plasmids (*ERG11*^WT^-pBSS2, *ERG11*^VF125AL^-pBSS2, *ERG11*^Y132F^-pBSS2, and *ERG11*^K143R^-pBSS2) were then used to generate transformation repair templates using a PCR primers set which introduced 50 bases of microhomology downstream of the *ERG11* open reading frame. Cas9-RNP were then constructed using crRNA which target unique sequences immediately up and downstream of the *ERG11* open reading frame, and transformations were performed by electroporation to generate transformants which were confirmed by Sanger sequencing to possess each of the four *ERG11* alleles of interest as previously described (15, 16). Additionally, transformants were confirmed to possess a single copy of *ERG11* by qPCR as previously described (15).

**FIG 1:**
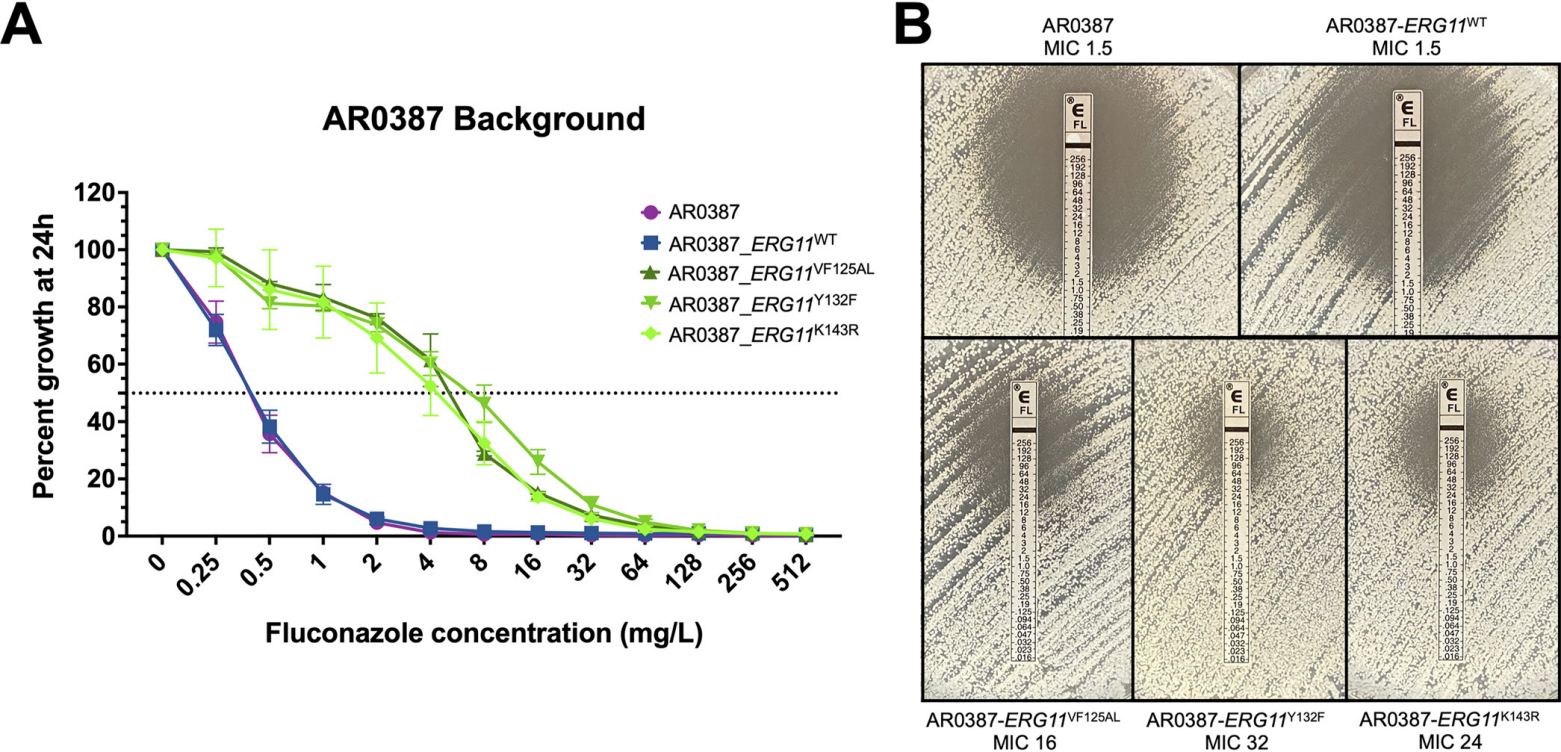
Introduction of *ERG11* mutations to the AR0387 background increases fluconazole resistance. (A) Fluconazole susceptibility of AR0387 and derivative *ERG11* strains as assessed by broth microdilution at 24 h. The relative percent growth as measured by absorbance at OD_600_ was determined comparing each strain or isolate to the corresponding untreated controls. The dotted horizontal line corresponds to 50% growth inhibition. Error bars represent the standard deviations of readings from three independent measurements of technical replicates from a single biological replicate. (B) Fluconazole susceptibility of AR0387 and derivative *ERG11* strains as assessed by Etest at 24 h.

**FIG 2:**
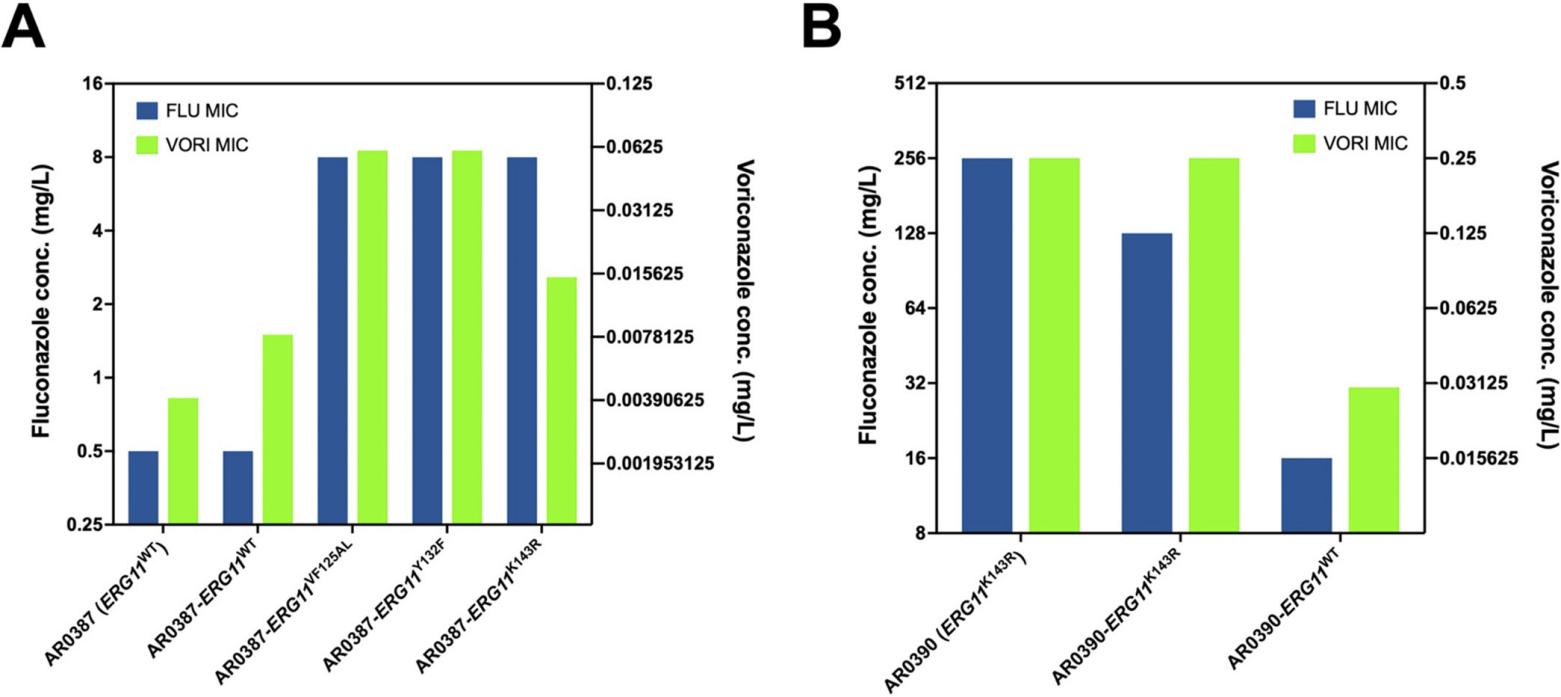
Mutations in C. auris *ERG11* confer comparable increases in fluconazole and voriconazole resistance. (A) Comparison of fluconazole and voriconazole susceptibility of AR0387 and derivative *ERG11* strains as assessed by broth microdilution at 24 h. (B) Comparison of fluconazole and voriconazole susceptibility of AR0390 and derivative *ERG11* strains as assessed by broth microdilution at 24 h. FLU, fluconazole; VORI, voriconazole.

The parental clinical isolate AR0387 and each of the derivative strains were then subjected to broth microdilution susceptibility testing using CLSI methods with minor modification as previously described, and the MIC for the five clinically available triazoles were determined (15, 16). Introduction of each of the three resistance-associated alleles (*ERG11*^VF125AL^, *ERG11*^Y132F^, and *ERG11*^K143R^) into the AR0387 background resulted in an 16-fold increase in fluconazole MIC relative to the parental isolate (Fig. 1A). This increase in fluconazole resistance was also observed by Etest, with the strain harboring the *ERG11*^Y132F^ allele exhibiting a slightly more prominent change in the zone of inhibition on solid media (Fig. 1B). Importantly, the *ERG11*^WT^ manipulation control strains exhibited no change in fluconazole MIC relative to the parental AR0387 (MIC 0.5 mg/L). Changes in voriconazole MIC were observed to be consistent with those observed in fluconazole MIC and increased by 4- to 16-fold for all strains with resistance-associated *ERG11* alleles (Fig. 2A). However, MIC for isavuconazole, itraconazole, and posaconazole were relatively unchanged (1 dilution or less) upon introduction any of the *ERG11* alleles into AR0387 (Table 1).

**TABLE 1:** Triazole antifungal MIC of *C. auris* clinical isolates and derivative *ERG11* strains as assessed by broth microdilution at 24 h^*a*^

Clinical isolate or Strain	Antifungal MIC (mg/L)				
	FLU	VORI	ISAVU	ITRA	POSA
AR0387 (*ERG11*^WT^)	0.5	≤0.004	≤0.001	0.008	0.008
AR0387-*ERG11*^WT^	0.5	0.008	≤0.001	0.008	0.004
AR0387-*ERG11*^VF125AL^	8	0.06	0.002	0.008	0.008
AR0387-*ERG11*^Y132F^	8	0.06	0.002	0.008	0.008
AR0387-*ERG11*^K143R^	8	0.015	0.002	0.015	0.008
AR0390 (*ERG11*^K143R^)	**256**	0.25	0.03	0.06	0.015
AR0390-*ERG11*^K143R^	**128**	0.25	0.03	0.06	0.03
AR0390-*ERG11*^WT^	16	0.03	0.015	0.03	0.015

a WT, wild type; FLU, fluconazole; VORI, voriconazole; ISAVU, isavuconazole; ITRA, itraconazole; POSA, posaconazole; MIC shown in bold exceed tentative CDC breakpoints.

**FIG 3:**
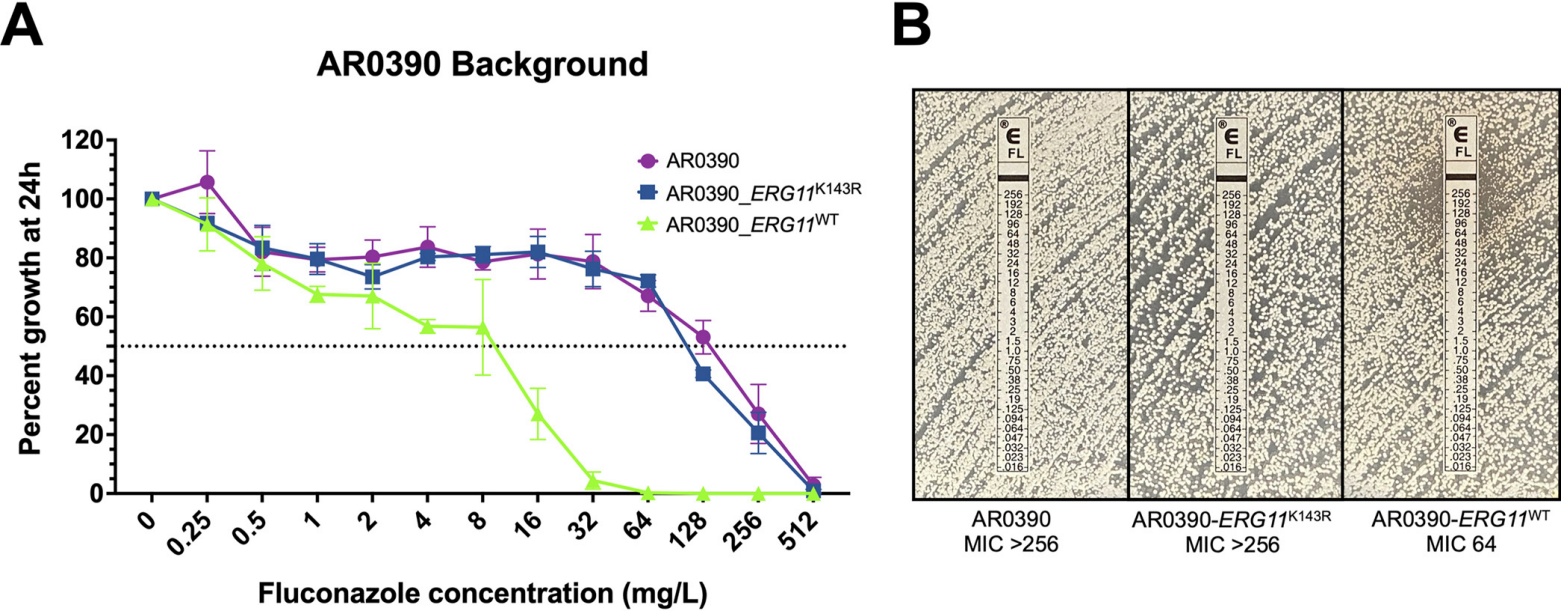
Correction of the *ERG11* mutation encoding the K143R amino acid substitution in the AR0390 background decreases fluconazole resistance. (A) Fluconazole susceptibility of AR0390 and derivative *ERG11* strains as assessed by broth microdilution at 24 h. The relative percent growth as measured by absorbance at OD_600_ was determined comparing each strain or isolate to the corresponding untreated controls. The dotted horizontal line corresponds to 50% growth inhibition. Error bars represent the standard deviations of readings from three independent measurements of technical replicates from a single biological replicate. (B) Fluconazole susceptibility of AR0387 and derivative *ERG11* strains as assessed by Etest at 24 h.

As confirmation, the complementary experiment was performed using the same methodology to correct the mutation encoding the K143R amino acid substitution in *ERG11* in highly fluconazole-resistant (MIC 256 mg/L) clinical isolate AR0390 (also known as B11205). This isolate was selected as it has been previously characterized and was recently utilized to construct the reference genome assembly for Clade Ic isolates, the vast majority of which harbor this single resistance-associated *ERG11*^K143R^ allele (15–17). The resulting *ERG11*^WT^ transformants, were observed to exhibit an 16-fold decrease in fluconazole MIC relative to the parental AR0390 (MIC 256 mg/L) (Fig. 3A). Conversely the manipulation control strain, still harboring the *ERG11*^K143R^ allele, was found to have only a single dilution reduction in fluconazole MIC (128 mg/L) by broth microdilution, with no appreciable change in the zone of inhibition observed by Etest (Fig. 3B). Moreover, an 8-fold decrease in resistance to voriconazole was observed upon introduction of the *ERG11*^WT^ allele, again following the changes observed with fluconazole MIC (Fig. 2B). However, consistent with the observations in the AR0387 background, MIC for isavuconazole, itraconazole, and posaconazole were largely unchanged (Table 1).

## DISCUSSION

The triazoles are the most commonly relied upon class of antifungals worldwide, accounting for more than 80% of all antifungal prescribing in the United States (18). These agents are widely utilized due to their relative low cost, availability in various formulations, and a generally favorable adverse effect profile. The triazoles exert antifungal activity through the competitive inhibition of sterol-demethylase, which catalyzes a critical and rate-limiting step in the ergosterol biosynthesis pathway (19). Ergosterol is the prominent membrane sterol among most pathogenic fungi, and inhibition of sterol-demethylase by the triazoles depletes cellular ergosterol and leads to the accumulation of other sterol precursors, including those thought to be toxic to the fungal cell.

Among pathogenic fungi, the role of mutations in the gene encoding sterol-demethylase in antifungal resistance has been most comprehensively studied in C. albicans, where a wide repertoire of mutations have been observed among fluconazole-resistant isolates, the majority of which are found within three hot spot regions corresponding to amino acids 105 to 165, 266 to 287, and 405 to 488 (4, 5). Two mutations affecting residues residing within the first of these three hot spots, encoding the Y132F and K143R substitutions, have been shown to greatly contribute to fluconazole resistance in C. albicans. The Y132 position is within the predicted catalytic site of sterol-demethylase and the Y132F substitution is predicted to interfere with fluconazole binding. The K143 position resides in a region proximal to the heme presumed to be involved in electron transfer from the P450 reductase and the K143R substitution affects catalytic efficiency (4). In C. albicans the Y132F substitution resulted in an 8-fold increase in fluconazole MIC whereas the K143R substitution resulted in a 16-fold increase. Both affected voriconazole MIC to a lesser extent (2-fold increase), the K143R substitution increased itraconazole MIC 2-fold, and neither had an effect on posaconazole susceptibility (5). To date the VF125AL double residue substitution has not been described in C. albicans. However, a single mutation resulting in the F126L substitution was found in a highly fluconazole-resistant C. albicans clinical isolate in conjunction with mutations encoding the E266D, S405F, and V437I substitutions (20). While the potential contribution to triazole resistance of this F126L-encoding mutation in C. albicans has not been delineated, the F126 residue is predicted to reside near the ligand access channel and modifications to this residue may interfere with the binding of sterol-demethylase inhibitors (20).

In C. albicans, mutations in *ERG11* often accompany activating mutations in *UPC2* which encodes the major transcriptional regulator of ergosterol biosynthesis (5). Such activation of *UPC2* leads to increased expression of genes of the ergosterol biosynthesis pathway including *ERG11* which in combination with *ERG11* mutations has a combinatorial effect on triazole susceptibility (5, 21, 22). To date, activating mutations in the C. auris ortholog of *UPC2*, B9J08_000270, alone or in combination with *ERG11* mutations have not been observed in clinical isolates.

While it cannot be denied that prior study of resistance-associated mutations in C. albicans *ERG11* have been informative of the role of sterol-demethylase mutations in other species of *Candida*, it is also imperative to acknowledge that species specific variations in the sterol-demethylase peptide sequences may have significant impact on triazole susceptibility. The predicted peptide sequence encoded by C. auris *ERG11* (B8441 reference sequence) shares 70.6% identity with that of C. albicans. Of the 156 residues which differ between these species, 46 reside within one of the three *ERG11* hot spot regions. Intriguingly, six of the variations within these three regions (F105, D116, D153, E266, R267, and V437), occur at residues that have been implicated in triazole resistance in C. albicans previously (Table 2). Moreover, five of the specific amino acids in wild type (B8441) C. auris that vary from wild type C. albicans (SC5314) have also been observed in either fluconazole-resistant isolates of C. albicans, or are naturally occurring variations shared between C. auris and C. krusei or C. glabrata, both of which have greater intrinsic resistance to fluconazole than C. albicans. Thus, it is tempting to speculate that in addition to the three C. auris *ERG11* mutations characterized in this work, these species-specific variations may also contribute to the greatly elevated fluconazole resistance in C. auris. Furthermore, these inherent variations also provide a potential explanation for the notably greater increase in voriconazole resistance conferred by the Y132F and K143R-encoding mutations in C. auris, relative to that reported in C. albicans.

**TABLE 2:**
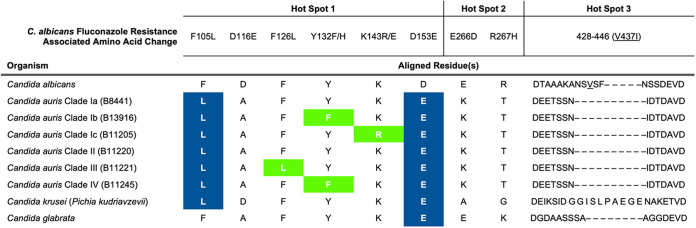
*ERG11* hot spot region amino acid variations between C. auris, C. albicans, C. krusei, and C. glabrata^*a*^

a Amino acid resides within *ERG11* hot spot regions which are associated with fluconazole resistance in *C. albicans* and which vary between *C. auris* and *C. albicans* are shown as compared to the corresponding residues in *C. krusei* and *C. glabrata*. Amino acid variations associated with fluconazole resistance in *C. auris* and studied in this work are shown in bold and highlighted in green. Additional amino acid varying between the *Candida* species shown which match variations associated with resistance in *C. albicans* are shown in bold and highlighted in blue. The length of peptide sequence corresponding to hot spot 3 in *C. albicans* varies among the *Candida* species shown. The *C. albicans* V437 residue in this region is shown underlined.

It is important to note that none of the three mutations tested here when introduced into the fluconazole-susceptible isolate AR0387 resulted in clinical fluconazole resistance as defined by the tentative breakpoints set forth by the CDC (11). Likewise, while correction of the K143R substitution to the wild type sequence in resistant clinical isolate AR0390 did confer susceptibility to fluconazole, the MIC remained high (16 mg/L) compared with the susceptible isolate AR0387 (0.5 mg/L). AR0390 also carries a mutation (encoding the A640V substitution) in the gene encoding the transcription factor Tac1B which likely leads to overexpression of the Cdr1 drug efflux transporter (15, 16). Correction of this mutation to the wild type sequence in clinical isolate AR0390 likewise confers susceptibility to fluconazole with an MIC of 16 mg/L. It is therefore likely that the high level of fluconazole resistance observed in this isolate is due to the combined effects of these mutations. Indeed, most fluconazole-resistant C. auris isolates studied to date carry mutations in both *TAC1B* and *ERG11* (17).

We have shown here for the first time the specific impact of the three most common mutations observed in *ERG11* on the susceptibility of C. auris to the commonly prescribed triazole antifungals. While all three of these mutations contribute to fluconazole resistance, none alone are sufficient to confer clinical resistance. Moreover, with each of the three *ERG11* mutations, voriconazole MIC were observed to increase comparably to fluconazole MIC. This is in stark contrast to isavuconazole, itraconazole, and posaconazole. Unlike fluconazole and voriconazole, the lipophilic side chains of all three of these triazoles are believed to interact with the sterol-demethylase enzyme at additional residues along the enzyme ligand access channel. As the three C. auris *ERG11* mutations studied in this work alter residues predicted to be at or near the catalytic domain of Erg11, these additional interactions along the ligand access channel may contribute to the maintained activity of isavuconazole, itraconazole, and posaconazole. Furthermore, as these data demonstrate that the activity of these triazoles are impacted to a much lesser extent by the predominant fluconazole-resistance mechanism identified among clinical isolates of C. auris, this may suggest a possible role for these triazoles in the treatment of fluconazole-resistant C. auris infections.

## MATERIALS AND METHODS

### Isolate, strains, and growth media used in this study.

Clinical isolates AR0387 (also known as B8441) and AR0390 (also known as B11205) were obtained from the CDC as part of the Antibiotic Resistance Isolate Bank program. Strains that were derived from these isolates as part of this study are listed in Table S2. Strains and isolates were routinely propagated in YPD (1% yeast extract, 2% peptone, 2% dextrose) medium and on YPD agar.

### Cas9-Ribonucleoprotein mediated transformations.

Strains were constructed using a Cas9-mediated allele swap using the *SAT-FLP* cassette as previously described (15). Briefly, *ERG11* alleles were amplified from AR0387 (*ERG11*^WT^ allele), AR0383 (VF125AL allele), UTHSCA DI-19-24 (Y132F allele), and AR0390 (K143R allele) using primers CAU_ERG11_ORF-F and CAU_ERG11_ORF-R (Table S1) and individually cloned into the pBSS2 plasmid using the restriction enzymes *SacII* and EagI. Notably, the *ERG11*^VF125AL^ and *ERG11*^Y132F^ alleles additionally include polymorphisms not associated with fluconazole resistance and common to clinical isolates of Clade III (nucleotide polymorphisms not encoding additional amino acid alterations) and Clade IV (encoding the amino acid substitutions K177R, N335S, and E343D), including fluconazole-susceptible isolates. Each of the four resulting plasmids (*ERG11*^WT^-pBSS2, *ERG11*^VF125AL^-pBSS2, *ERG11*^Y132F^-pBSS2, and *ERG11*^K143R^-pBSS2) were then used to generate transformation repair templates comprised of the cloned *ERG11* allele and *SAT-FLP* cassette using a PCR primer set which introduced 50 bases of microhomology downstream of the *ERG11* open reading frame. Cas9-RNP were then constructed using crRNA which target unique sequences immediately up and downstream of the *ERG11* open reading frame. Transformations were performed by electroporation, the excision of the *SAT-FLP* cassette from the targeted locus was induced, and positive transformants were confirmed by Sanger sequencing as previously described (15).

### MIC determination.

A modified CLSI MIC determination employing the standard endpoint of 50% growth inhibition was performed using broth microdilution in biological duplicate, each with three technical replicates, as previously described, and MIC from biological replicates were in agreement (15). MIC determination by ellipsometer test (Etest) were performed using the manufacturer’s (bioMérieux USA, Chicago, IL) instructions with modifications per the Clinical and Laboratory Standards Institute document M44-A2.
