# Supplementary material for: Delineation of the Direct Contribution of Candida auris ERG11 Mutations to Clinical Triazole Resistance
Source: Microbiol Spectr. 2021 Dec 8;9(3):e01585-21. doi: 10.1128/Spectrum.01585-21 (PMC8653815; doi:10.1128/Spectrum.01585-21)
Supplement: SUPPLEMENTAL FILE 1 — Supplemental material. Download SPECTRUM01585-21_Supp_1_seq9.pdf, PDF file, 0.5 MB [file spectrum01585-21_supp_1_seq9.pdf]

SUPPLEMENTARY TABLES

Table S1. Primers used in these studies.

Primer Name	Sequence[1]	Description
CAU_ERG11_ORF-F	TCACCGCGGGAGTCGTCGCGTAAATACAATGCC	ORF amplification with *SacII* site
CAU_ERG11_ORF-R	TCACGGCCGGAACCTAGTCCTCTTCCAAGCGG	ORF amplification with *EagI* site
CAU_ERG11_RT_F	CGACTTGAGTTCAACATTTTCACTCGC	Repair template amplification
	CAAGCAATAGTCAAGCATTCTTTCCTTTGAGAGTT	Repair template amplification
CAU_ERG11_RT_R	GAAGCTGTCCAATTTCGTTGCCCAACATCACTCCG	
	GGTTTTCCCAGTCACGACGT	
CAU_ERG11_5'_crRNA	AAAAGAACCTTTGACAGCTA	5' RNP target sequence
CAU_ERG11_3'_crRNA	TATTGTGCACTCTACGCATA	3' RNP target sequence
CAU0130K02m	CCATCGTATAGTGGTCCTCC	forward sequencing primer 1
CAU0131K02m	CGTTGGCCACGCCGTTG	reverse sequencing primer 3
CAU0132K02m	GGGACTTGATCGACTCGC	forward sequencing primer 2
CAU0133K02m	GGCATGTGCAAACGCAACG	reverse sequencing primer 2
CAU0134K02m	GGCGGTGGTAGACACCG	forward sequencing primer 3
CAU0135K02m	CTGGCCTGAGTCACCAC	reverse sequencing primer 1

1. Sequences are listed in 5' to 3' orientation, and restriction enzyme recognition sites are denoted by underlined nucleotides

Table S2. Isolates and strains used in these studies.

Isolate/ Strain Name	*ERG11* sequence
AR0387	WT
AR0387_*ERG11*-WT-2-34	WT
AR0387_*ERG11*-VF125AL-8-11	VF125AL
AR0387_*ERG11*-Y132F-5-13	Y132F
AR0387_*ERG11*-K143R-3-10	K143R
AR0390	K143R
AR0390_*ERG11*-K143R-2-6	K143R
AR0390_*ERG11*-WT-7-15	WT
